# Gender differences in fasting and postprandial metabolic traits predictive of subclinical atherosclerosis in an asymptomatic Chinese population

**DOI:** 10.1038/s41598-022-20714-6

**Published:** 2022-10-07

**Authors:** Xinpeng Loh, Lijuan Sun, John Carson Allen, Hui Jen Goh, Siew Ching Kong, Weiting Huang, Cherlyn Ding, Nabil Bosco, Leonie Egli, Lucas Actis-Goretta, Faidon Magkos, Fabrizio Arigoni, Khung Keong Yeo, Melvin Khee-Shing Leow

**Affiliations:** 1 Duke-NUS Medical School, 30 Medical Drive, Singapore, 117609 Singapore; 2 Singapore Institute for Clinical Sciences, Singapore, Singapore; 3 National Heart Center Singapore, Singapore, Singapore; 4 Nestlé Research Singapore Hub, Singapore, Singapore; 5 Nestlé Institute of Health Sciences, Nestlé Research, Lausanne, Switzerland; 6 University of Copenhagen, Frederiksberg, Denmark; 7 Department of Endocrinology, Tan Tock Seng Hospital, Singapore, Singapore; 8 Lee Kong Chian School of Medicine, Nanyang Technological University, Singapore, Singapore

**Keywords:** Cardiovascular biology, Cardiology, Biomarkers, Predictive markers

## Abstract

The prediction utility of Framingham Risk Score in populations with low conventional cardiovascular risk burden is limited, particularly among women. Gender-specific markers to predict cardiovascular risk in overtly healthy people are lacking. In this study we hypothesize that postprandial responses triggered by a high-calorie meal test differ by gender in their ability to triage asymptomatic subjects into those with and without subclinical atherosclerosis. A total of 101 healthy Chinese subjects (46 females, 55 males) at low risk of coronary heart disease completed the study. Subjects underwent cardiovascular imaging and postprandial blood phenotyping after consuming a standardized macronutrient meal. Prediction models were developed using logistic regression and subsequently subjected to cross-validation to obtain a de-optimized receiver operating characteristic (ROC) curve. Distinctive gender differences in postprandial trajectories of glucose, lipids and inflammatory markers were observed. We used gender-specific association with different combinations of postprandial predictors to develop 2 models for predicting risk of subclinical atherosclerosis in males (ROC AUC = 0.7867, 95% CI 0.6567, 0.9166) and females (ROC AUC = 0.9161, 95% CI 0.8340, 0.9982) respectively. We report novel postprandial models for predicting subclinical atherosclerosis in apparently healthy Asian subjects using a gender-specific approach, complementing the conventional Framingham Risk Score.

*Clinical Trial Registration*: The trial was registered at clinicaltrials.gov as NCT03531879.

## Introduction

Cardiovascular disease (CVD) is responsible for a significant proportion of morbidity and mortality in Singapore over the last three decades^1,2^. In 2019 alone, nearly 1 in 3 deaths in Singapore was caused by CVD^1^. This results in a smaller workforce and incurring greater healthcare expenditure, subsequently taxing the Singapore economy^3^. To mitigate disease burden, there are contemporary prediction tools that provide differential and individualized clinical insights into CVD development. The likelihood of future cardiovascular events in asymptomatic individuals is typically estimated using conventional cardiovascular risk factors (CVRF) such as cigarette smoking, diabetes status, hyperlipidemia, and hypertension^4^.

The Framingham Risk Score (FRS) epitomizes an important advancement in coronary heart disease (CHD) prevention. FRS considers CVRFs, age and gender to predict the 10-year risk of developing CHD and is the most common clinical metric used to identify high-risk individuals^5^. While this assessment is well validated in elderly people and in men, there is increasing evidence of subclinical atherosclerosis (SA) and CVD even in populations with low conventional CVRF burden^6,7^. Hence, FRS has limited predictive value to forecast CHD incidence in low FRS (< 10%) populations, particularly in younger adults and women^8^.

Past epidemiological studies reported that symptomatic atherosclerosis typically manifests a decade later in women compared to men, while myocardial infarction incidents are delayed by approximately 5–10 years^9^. Although much of this cardio-protection is attributed to the beneficial effects of estrogen, the failure of hormone replacement therapy to decrease CHD highlights the complex relationship shared between atherosclerosis process and biological sex^10^. While FRS is known to underestimate risk of CHD in women^8^, there is a lack of improved gender-specific risk stratification strategies to supplement the FRS. Even though the Framingham risk equation factors in gender, it often classifies asymptomatic women as being low-risk, even in the presence of significant coronary artery disease^8^. The Reynolds Risk Score (RRS) was specifically developed for women and helped reclassify those initially deemed to be low risk using the FRS model. However, direct comparisons of the two models proved the RRS to be a more accurate short-term predictor but with modest overall gains^11^.

Imaging remains the gold standard for detection of SA, either via carotid intima-media thickness (IMT) measurements^12^ or carotid artery plaque detection^13^. Coronary artery calcification (CAC) score used to quantify the degree of carotid artery plaque has demonstrated incremental prognostic value over conventional CVRFs^14,15^, especially in females with a low FRS^16^. Despite its promise, the logistical burden and other pragmatic considerations have restricted routine use of imaging for SA detection in daily practice^6^.

Current guidelines do not consider healthy individuals with no CVRFs for preventive measures^17^. Therefore, there is an increasing need to discover improved markers for predicting SA, especially given the paradoxical observation of women experiencing cardiovascular events despite being deemed to be at low short-term risk^8,18^.

Growing evidence has identified several postprandial biomarkers with notable ability to predict the presence of SA, suggesting potential for use in developing improved surveillance models of CVD. Prior work has shown that consumption of a mixed meal test can significantly strengthen the association of triacylglycerol^19^, glucose^20^, and inflammatory markers^21^ with future cardiovascular event occurrence. Postprandial triacylglycerol concentrations^19^ and hyperglycemia^20^ were demonstrated to be better predictors of CVD risk than their fasting equivalents. Several reports have also shown that postprandial glycemia may be an independent risk marker for CVD. Use of a high-calorie mixed meal test perturbs the metabolic system and likely accentuates subclinical abnormalities that are otherwise masked in the fasting state. Furthermore, a standardized mixed meal test was reported to trigger wide ranging and individually unique postprandial responses amongst subjects^22^, indicating that postprandial biomarkers could gear progress towards personalized interventions. Therefore, our study aimed to assess the hypothesis that postprandial biomarker responses initiated by a controlled mixed meal test can effectively triage asymptomatic individuals into those with and without SA, in a gender-specific manner.

## Subjects and methods

### Study design

The study took place across two sites: National Heart Center of Singapore (NHCS) for cardiovascular measurements, and the Singapore Institute for Clinical Sciences (SICS), A*STAR for the mixed-meal test. Non-invasive imaging was used to evaluate presence of SA in 101 healthy Chinese adults recruited between May 2018 and June 2019. Inclusion criteria were the following: (1) be willing and able to sign written informed consent in English or Chinese prior to trial entry; (2) age 40–54 years; (3) male or female; (4) Chinese ethnic group (having both grandparents Chinese); (5) low Framingham risk of CAD (< 10%); (6) apparently healthy, based on clinical judgement. Exclusion criteria were: (1) food allergy to any of the constituents of the meal challenge (milk proteins, soy, or lactose, including lactose intolerance); (2) not willing or able to comply with scheduled visits and the requirements of the study protocol; (3) contraindication to MRI (i.e., cardiac pacemaker, brain aneurysm or clips, electronic implants or prosthesis, and others); (4) pregnant or lactating women, based on clinical judgement; (5) women on hormonal replacement therapy, oral/injection/transdermal hormonal contraceptive, or hormonal intrauterine device; (6) morbid obesity (BMI ≥ 40, measured in kg/m^2^); (7) previous myocardial infarction; (8) known CAD–prior coronary revascularization; (9) known documented peripheral arterial disease; (10) previous stroke (defined as new focal neurological deficit persisting > 24 h); (11) use of antihypertensive agents; (12) prior history of cancer (excludes precancerous lesions); (13) life expectancy < 1 year; (14) known definite diabetes mellitus or on treatment for diabetes mellitus, autoimmune disease, or genetic disease, or endocrine and metabolic diseases, including hyperlipidemia; (15) psychiatric illness; (16) asthma or chronic lung disease requiring long-term medications or oxygen; (17) chronic infective disease, including tuberculosis, hepatitis B and C, and HIV; and (18) currently participating or having participated in another clinical trial within 4 weeks prior to trial start. Subjects underwent clinical interviews, clinical laboratory analyses, vascular imaging, and fasting and postprandial blood phenotyping. The study protocol was approved by SingHealth Centralised Institutional Review Board (2018/2116) and all eligible participants have provided written informed consent. This trial was registered at ClinicalTrials.gov (NCT03531879). All procedures were ethically conducted in accordance with the Declaration of Helsinki.

### Definition of low FRS

Subjects participating in this study had low risk (≤ 10%) of developing CHD, as defined by the Framingham Heart Study CHD 10-year risk score^23^. Blood pressure, smoking status, total cholesterol, low-density lipoprotein cholesterol (LDL-C) and high-density lipoprotein cholesterol (HDL-C) levels at fasting state were used to calculate the 10-y risk score of CHD.

### Cardiovascular assessments

Presence of atherosclerotic plaque in bilateral carotid arteries, bilateral iliofemoral arteries and abdominal aorta, was determined by duplex ultrasound (Philips EPIQ-7G Ultrasound system and iU22-Ultrasound system 795050). Plaques were defined as a focal protrusion into the arterial lumen of thickness > 0.5 mm or > 50% of the surrounding intima-media thickness or a diffuse thickness > 1.5 mm measured between the media-adventitia and intima-lumen interfaces^24^. Coronary artery calcification (CAC) was measured by a non-contrast chest CT scan to compute the Agatston score (Canon Scanner Aquilion-One, Genesis-Edition, Model:TSX-305A/55)^25^. SA was defined as presence of atherosclerotic plaque in either the carotid, abdominal aortic or iliofemoral territory and/or a CAC Agatston score ≥ 1. Participants with 0 vascular sites affected were classified as disease free (plaque = 0). Participants with at least 1 plaque site affected (plaque > 0) were considered to have SA.

### Blood sampling

Within 2 weeks from the cardiovascular imaging assessment, subjects visited the SICS for the mixed-meal test. Blood biomarkers were measured after 10 h of fasting, and at frequent intervals for 6 h after consuming the test meal. Venous blood samples were drawn into precooled sterile tubes (Vacutainer, Becton Dickinson) containing K2EDTA (final concentration 1.8 mg/mL) and protease inhibitor cocktail (A.G. Scientific, Inc, CA, USA) for plasma samples, and sterile tubes containing silica clot activator for serum samples. Plasma was recovered by low-speed centrifugation (1750×*g*, 10 min, 4 °C).

Venous blood was sampled at fasting and at 10, 20, 30, 45, 60, 90, 120, 240 and 360 min after consumption of the test-meal for determination of insulin, glucose and C-peptide, due to the acute insulin response in which over 90% of the pancreatic beta cell response occurs within the initial 10–30 min. Blood samples were sampled at fasting and at 60, 120, 240 and 360 min for determination of apolipoprotein B48, leptin, adiponectin, C-Reactive Protein (CRP), Tumor Necrosis Factor Alpha (TNFα), Interleukin 6 (IL-6), Plasminogen activator inhibitor-1 (PAI-1), vascular cell adhesion molecule 1 (VCAM-1), Intercellular Adhesion Molecule 1 (ICAM-1), E-selectin, LDL-C, HDL-C, total cholesterol, and triacylglycerol. Plasma glucose concentration was determined using the glucose oxidase method on an automated glucose analyzer (YSI 2300 Stat Plus; YSI Life Sciences, Yellow Spring, OH, USA). Plasma insulin and C-peptide concentrations were determined by electrochemiluminescence technology (Roche/ Hitachi cobas e411 immunochemistry analyzer; Roche Diagnostics, Indianapolis, IN). Apolipoprotein B48, leptin, adiponectin, CRP, TNFα, IL-6, PAI-1, VCAM-1, ICAM-1, and E-selectin were determined using a 6-plex Human Luminex Screening Human Magnetic Micro-bead Immunoassay (R&D Systems, Inc.), a 3-Plex Luminex Screening Human Magnetic Assay (R&D Systems, Inc.) and a Human Apolipoprotein B48 ELISA Kit (Elabscience Biotechnology Inc.). Data analysis was done on Bio-Plex Manager™ 6.1.1 (Bio-Rad). Standard curves were generated with a 5-parameter logistic algorithm, reporting values for both mean florescence intensity and concentration data.

### Mixed-test meal

The test meal consisted of 237.5 mL of a nutritional drink (Nestle Heath Sciences, Lausanne, Switzerland) mixed with 100 mL of commercially available whipping cream. This composition was determined based on a systematic review that defined an optimal nutritional stress test^26^: 75 g glucose, 60 g palm olein and 20 g dairy protein served in a ~ 337 mL liquid meal providing a total of ~ 930 kcal.

### Statistical analyses

Statistical comparisons between participants with and without SA were performed using Fisher’s exact test for categorical variables and Student’s *t* test for comparing two independent samples in the evaluation of continuous variables. Univariate and multivariable logistic regression models incorporating a stepwise variable selection approach for model building were used to analyze the associations of multiple covariates with the presence of SA and select predictors.

To estimate the biomarker response during the entire 6-h postprandial period, the incremental AUC relative to the fasting level was calculated. The associations between metabolic markers and presence of atherosclerosis were then assessed using univariable logistic regression. Variables that showed a marginal univariate association with *p* < 0.10 with the atherosclerotic plaque category were included as candidate predictors in the multivariable analysis incorporating the stepwise selection algorithm. Variable selection in the development of predictive models can encompass both statistical and clinical considerations. We set *P* < 0.10 as a less stringent criteria so that additional variables could qualify as candidate predictors for the stepwise multivariate selection on the basis of scientific relevance and/or clinical experience. Multivariable models were subsequently generated by multiple stepwise linear regression analysis to identify parsimonious subsets of variables predictive of SA. Stepwise selection significance levels were set at *p* = 0.10 to enter and stay. ROC curves were obtained from multivariable logistic regression models, and the Youden index was used to find the statistically optimal threshold for prognosticating high versus low risk. The statistically optimal threshold for males was a predicted probability of *P* = 0.39 corresponding to the Youden index of J = 0.727; for females, *P* = 0.55, corresponding to J = 0.832; and for the grouped samples, *P* = 0.35, and J = 0.543. Predictive models with corresponding ROC curve summaries, AUCs, predictive statistics, optimal cut-points discrimination capabilities are summarized in the figures and tables below. Predictive models were then subjected to tenfold cross-validation to obtain a de-optimized ROC curve. We used the Delong-Delong approach to compare the ROC AUC between the Framingham score model and the postprandial biomarker predictive model. Cross-validation was performed by invoking the CROSSVALIDATE option of the SAS logistic regression procedure (PROC LOGISTIC) which requests the cross validated individual predicted probability of each response level. These probabilities are derived from the leave-one-out principle—that is, dropping the data of one subject and re-estimating the parameter estimates. However, PROC LOGISTIC uses a computationally less expensive one-step approximation to compute the parameter estimates which is valid for binary response models. All statistical analyses were performed using the SAS University Edition (SAS Institute Inc., NC, USA). Unless otherwise stated, statistical significance was set at *p* < 0.05.

## Results

### Clinical characteristics of subjects

A total of 116 healthy middle-aged subjects consented for the study, but 11 participants who did not meet the inclusion criteria were excluded. A further 4 subjects later dropped out of the study, leaving 101 subjects who completed all assessments (46 females, 55 males). Gender distribution of demographic characteristics and baseline metabolic indicators are presented in Table 1. The male participants were 47.53 ± 4.05 years old and had a mean FRS 5.56 ± 2.52, while female participants were 47.85 ± 4.54 years old and had a mean FRS 5.76 ± 2.97. This translates into an average 10-year CHD risk score of ≤ 3% for men and ≤ 1% for women across 3 major racial groups in Singapore^27^. Despite the absence of conventional CVRFs, at least 1 atherosclerotic site could be identified in 38 ostensibly healthy subjects (37.6%) (Supplementary Table 1). There were marginal differences in the vascular distribution of subclinical atherosclerosis between males and females (Table 2).

**Table 1:** Demographic and clinical characteristics at baseline (fasting state) for males versus females.

Variable	Males (n = 55)	Females (n = 46)	Mean difference (CI)	*P* value
**Gender**				
** Male**				
** Female**				
BMI (kg/m^2^)	23.77 ± 2.82	23.18 ± 2.91	− 0.59 (− 1.72, 0.55)	0.3081
Weight (kg)	70.45 ± 9.59	59.05 ± 7.74	− 11.39 (− 14.88, − 7.91)	** < .0001**
Glucose (mmol/L)	5.11 ± 0.40	4.97 ± 0.36	− 0.14 (− 0.30, 0.0076)	0.0624
Insulin (μU/mL )	8.25 ± 5.61	8.0 ± 4.71	− 0.25(− 2.32, 1.82)	0.8132
c-peptide (ng/mL)	2.01 ± 0.85	1.97 ± 0.76	− 0.041 (− 0.36, 0.28)	0.7994
LDL-C (mmol/L)	3.35 ± 0.74	3.25 ± 0.73	− 0.10 (− 0.39, 0.19)	0.4934
HDL-C (mmol/L)	1.43 ± 0.30	1.67 ± 0.32	0.24 (0.12, 0.36)	**0.0002**
Triacylglycerol (mmol/L)	1.21 ± 0.67	0.88 ± 0.41	− 0.33 (− 0.55, − 0.12)	**0.0028**
Cholesterol (mmol/L)	5.33 ± 0.86	5.32 ± 0.88	− 0.014 (− 0.36, 0.33)	0.9343
Age	47.53 ± 4.05	47.85 ± 4.54	− 0.32 (− 1.38, 2.02)	0.7084
Framingham Score	5.56 ± 2.52	5.76 ± 2.97	0.20 (− 0.89, 1.28)	0.7188
HOMA-IR	2.05 ± 1.50	1.89 ± 1.16	− 0.16 (− 0.70, 0.38)	0.5615
HOMA-β	84.36 ± 54.66	93.52 ± 54.17	− 9.16 (− 12.42, 30.74)	0.4018
Adiponectin (pg/mL)	5,029,254 ± 2,137,308	6,058,005 ± 2,196,463	1,028,751 (− 170,674, 1,886,829)	**0.0193**
TNFα (pg/mL)	20.44 ± 15.73	12.78 ± 7.13	− 7.66 (− 12.64, − 2.67)	**0.0018**
PAI-1 (pg/mL)	29,484.4 ± 11,363.7	23,313.9 ± 9231.6	− 6170.6 (− 10,312.9, − 2028.2)	**0.0039**
Leptin (pg/mL)	2446.2 ± 2267.6	8701.9 ± 6189.6	6255.7 (4473.1, 8038.4)	** < .0001**

*P* value from 2-sample *t* test for continuous variables and Fisher’s exact test for categorical variables. BMI, Body mass index; LDL-C, Cholesterol in low-density lipoprotein; HDL-C, Cholesterol in high-density lipoprotein; HOMA-IR, Homeostasis model assessment of insulin resistance; HOMA-β, Homeostasis model assessment of β-cell function; TNFα, Tumor necrosis factor alpha; PAI-1, Plasminogen activator inhibitor-1. HOMA-IR was calculated by fasting plasma insulin (uU/mL) × fasting plasma glucose (mmol/L)/22.5. HOMA-β was calculated by 20 × fasting plasma insulin (uU/mL)/fasting plasma glucose (mmol/L)–3.5.

Significant values are in bold.

**Table 2:** Distribution of plaques amongst the various vascular territories stratified by gender.

Vascular distribution	Grouped	Males	Females	*P* value
Coronary arteries	23 (22.7%)	17 (30.91%)	6 (13.04%)	0.055
Carotid arteries	12 (11.88%)	7 (12.73%)	5 (10.87%)	1.000
Abdominal aorta	1 (0.99%)	1 (1.82%)	0	1.000
Ilio-femoral arteries	14 (13.86%)	8 (14.55%)	6 (13.04%)	1.000
*1 site involved*	28 (28%)	18 (33%)	10 (22%)	
*2 sites involved*	8 (8%)	6 (11%)	2 (4%)	
*3 sites involved*	2 (2%)	1 (2%)	1 (2%)	

*P* value represents the statistical difference between males and females at each vascular site.

### Interactions between baseline clinical indices and fasting biomarkers with gender and SA

First, we investigated any gender differential distribution of baseline clinical characteristics and fasting metabolites. As the FRS algorithm is known to have better accuracy in predicting CHD in men^8^, we also sought to determine if gender could be an effect modifier for SA status. Even though anthropomorphic parameters such as weight were significantly higher amongst males (Table 1), gender had no role in modifying its effect on SA status (Table 3). On the contrary, fasting metabolic biomarkers were significantly different between genders. Baseline HDL-C, adiponectin and leptin were higher in females, while baseline triacylglycerol, TNFα and PAI-1 were higher in males (Table 1). Amongst these, gender had significant interactions with HDL-C in altering SA status (Table 3). No gender differences were observed for fasting glucose, insulin, LDL-C, and total cholesterol. Age and FRS were significantly associated with the presence of SA in males only, while fasting cholesterol and leptin were significantly associated with the presence of SA in females only. However, we found no significant gender bias in the distribution of SA (Supplementary Table 1). These findings reaffirm fundamental gender differences to the early atherosclerosis process which therefore may require separate SA prediction models.

**Table 3:** Demographic and clinical characteristics at baseline (fasting state) for atherosclerotic plaque versus no atherosclerotic plaque groups stratified by gender.

Variable	Plaque 0		*Plaque* > *0*		*P* value for plaque category		*P* value for gender distribution	*P* value for gender* variable interaction on plaque category
	Males (n = 30)	Females (n = 33)	Males (n = 25)	Females (n = 13)				
BMI (kg/m^2^)	23.85 ± 2.80	23.26 ± 3.03	23.68 ± 2.89	22.98 ± 2.68		0.8630	0.3081	0.9331
Weight (kg)	70.21 ± 9.15	60.07 ± 8.25	70.74 ± 10.28	56.48 ± 5.77		0.6547	** < .0001**	0.1836
Glucose (mmol/L)	5.06 ± 0.39	5.0 ± 0.34	5.17 ± 0.40	4.88 ± 0.42		0.5818	0.0624	0.1410
Insulin (uU/mL )	7.47 ± 4.86	8.15 ± 4.79	9.19 ± 6.37	7.62 ± 4.66		0.4426	0.8132	0.3573
C-Peptide (ng/ml)	1.88 ± 0.79	2.02 ± 0.81	2.17 ± 0.90	1.84 ± 0.66		0.5379	0.7994	0.1900
LDL-C (mmol/l)	3.31 ± 0.74	3.13 ± 0.74	3.41 ± 0.75	3.58 ± 0.61		0.0942	0.4934	0.2667
HDL-C (mmol/L)	1.48 ± 0.28	1.62 ± 0.26	1.37 ± 0.31	1.80 ± 0.42		0.6128	**0.0002**	**0.0271**
Triacylglycerol (mmol/L)	1.13 ± 0.70	0.91 ± 0.43	1.31 ± 0.64	0.79 ± 0.34		0.3386	**0.0028**	0.2279
Cholesterol (mmol/l)	5.30 ± 0.86	*5.15* ± *0.89*	5.37 ± 0.87	*5.73* ± *0.72*		0.1318	0.9343	0.1907
age	*46.47* ± *4.15*	47.30 ± 4.64	48.80 ± 3.59	49.23 ± 4.13		**0.0188**	0.7084	0.6239
Framingham score	*4.83* ± *2.57*	5.42 ± 2.89	*6.44* ± *2.20*	6.62 ± 3.10		**0.0144**	0.7188	0.4227
HOMA-IR	1.84 ± 1.31	1.94 ± 1.20	2.30 ± 1.71	1.75 ± 1.08		0.4333	0.5615	0.3105
HOMA-β	78.66 ± 48.77	91.98 ± 50.74	91.19 ± 61.31	97.41 ± 64.15		0.4941	0.4018	0.7510
Adiponectin (pg/mL)	5,454,728 ± 2,361,782	6,099,140 ± 2,424,829	4,518,684 ± 1,743,636	5,953,585 ± 1,547,828		0.0853	**0.0193**	0.3452
TNFα (pg/mL)	23.50 ± 16.96	13.66 ± 8.02	16.77 ± 13.56	10.54 ± 3.38		0.1688	**0.0018**	0.3042
PAI-1 (pg/mL)	27,463.6 ± 11,895.7	21,808.6 ± 9059.3	31,909.4 ± 10,408.2	27,134.9 ± 8870.3		**0.0089**	**0.0039**	0.4767
Leptin (pg/mL)	2147.8 ± 1643.6	*9630.3* ± *6828.8*	2804.4 ± 2839.2	*6345.4* ± *3305.9*		**0.0363**	** < .0001**	0.0919

In italic are significant differences between plaque category within gender subgroups (*P < 0.05).

Significant values are in bold.

Body mass index (< 24.9 kg/m^2^), fasting glucose (< 6.0 mmol/L), fasting insulin (< 25 μU/mL), fasting HDL-C (> 1.0 mmol/L) and fasting triacylglycerol (< 1.70 mmol/L) in both genders were well below current thresholds to necessitate clinical intervention. Only LDL-C (> 3.30 mmol/L) in males and total cholesterol (> 5.20 mmol/L) in both genders were slightly above normal reference ranges (Table 1) but showed no significant differences when a between-plaque-category comparison was performed (Table 3). Indices of insulin resistance (HOMA-IR) and pancreatic beta cell function (HOMA-β)^28^ were uniformly distributed, and demonstrated early signs of insulin resistance (HOMA-IR > 1.8) and diminished beta cell function (HOMA-β < 100%) across both genders.

Next, we sought to evaluate if any metabolic biomarkers in the fasted state could be predictive of SA. We found that multivariable logistic regression models based on fasting biomarkers could not reliably predict SA. The best combination based on FRS and fasting PAI-1 achieved a ROC AUC of 0.704 with wide 95% CI (0.597, 0.811) (Supplementary Fig. 1).

## Association of postprandial indices with SA

We then appraised the prediction performance of postprandial biomarkers for SA. We first tested 564 features using univariate ROC curve analyses in the overall study sample (n = 101) and shortlisted candidate predictors with marginal association to SA (Supplementary Table 2). Even when pooled with postprandial biomarkers, FRS continued to emerge among the top candidates for predicting SA. Overall, PAI-1 concentration at 60 min post-meal was the top predictor of SA. Both fasting and postprandial levels of PAI-1 were consistently elevated in the SA group. This is congruent with previous studies^29^, but a magnified postprandial PAI-1 profile has not been reported.

### Association of gender stratified postprandial indices with SA

Subsequently, we stratified the dataset for a gender-specific analysis and found each gender to have distinctly different measures of association. TNFα, insulin, cholesterol and FRS were identified as independent predictors of SA in males (Table 4), while C-peptide, glucose and triacylglycerol were identified for females (Table 5). Supplementary Fig. 2 compares postprandial biomarker concentrations as a function of time between males and females. Each shortlisted biomarker had different post-meal trajectory and could distinguish SA status in a gender-specific manner.

**Table 4:** Potential candidate predictors of subclinical atherosclerosis in males (n = 55) assessed using univariable logistic regression.

Category	Variables	Univariate logistic regression		Multivariable logistic regression	
		OR (95% CI)	*P* value	OR (95% CI)	*P* value
Inflammatory	Diff TNFα Conc. t60–t120 min	0.629 (0.385, 1.026)	0.0635		
	**Diff TNFα Conc. t60**–**t240 min**	**0.622 (0.366, 1.058)**	**0.0800**	**0.511 (0.268, 0.975)**	**0.0418**
	TNFα iAUC t0–t240 min	0.997 (0.993, 1.000)	0.0700		
	TNFα iAUC t0–t360 min	0.998 (0.996, 1.000)	0.0619		
	Diff TNFα Conc. from fasting to Cmax	0.658 (0.407, 1.065)	0.0883		
Insulin sensitivity	Diff Insulin Conc. t0–t45 min	0.987 (0.972, 1.002)	0.0913		
	**Diff Insulin Conc. t30**–**t45 min**	**0.981 (0.96, 1.003)**	**0.0910**	**0.969 (0.943, 0.996)**	**0.0232**
	ln Adiponectin Conc. t120 min	0.242 (0.054, 1.079)	0.0628		
	ln Adiponectin Conc. t360 min	0.255 (0.055, 1.178)	0.0802		
Total cholesterol	Diff Cholesterol Conc. t0–t60 min	0.020 (< 0.001, 0.676)	0.0294		
	Diff Cholesterol Conc. t0–t360 min	0.077 (0.004, 1.600)	0.0976		
	Cholesterol iAUC t0–t60 min	0.807 (0.684, 0.953)	0.0115		
	**Cholesterol iAUC t0**–**t120 min**	**0.908 (0.842, 0.979)**	**0.0115**	**0.884 (0.805, 0.970)**	**0.0091**
	Cholesterol iAUC t0–t240 min	0.943 (0.898, 0.989)	0.0162		
	Cholesterol iAUC t0–t360 min	0.966 (0.940, 0.993)	0.0143		
	Diff Cholesterol Conc. from fasting to Cmax	0.008 (< 0.001, 0.427)	0.0172		
Demographic characteristics	Age	1.167 (1.010, 1.350)	0.0368		
	**Framingham Score**	**1.317 (1.041, 1.666)**	**0.0217**	**1.477 (1.088, 2.006)**	**0.0124**

Odds ratios are expressed per standard deviation increase in each continuous risk factor. Highlighted variables are independent risk predictors of subclinical atherosclerosis admitted by stepwise selection into the multivariable logistic regression models. OR, Odds ratio; CI, Confidence interval; TNFa, Tumor necrosis factor alpha; iAUC, incremental area under curve; Cmax, Peak concentration; Other demographic characteristics such as BMI, Diastolic blood pressure, Systolic blood pressure, waist circumference, weight, fasting glucose, fasting LDL-C, fasting HDL-C, fasting Triglyceride, fasting total cholesterol, age and Framingham score were also examined. Variables not listed in table have *p* > 0.10 in the univariate logistic regression models.

Significant values are in bold.

**Table 5:** Potential candidate predictors of subclinical atherosclerosis in females (n = 46) assessed using univariable logistic regression.

Category	Variables	Univariate logistic regression		Multivariable logistic regression	
		OR (95% CI)	*P* value	OR (95% CI)	*P* value
Insulin sensitivity	Diff Insulin Conc. t0–t60min	0.944 (0.903, 0.987)	0.0110		
	Diff Insulin Conc. t0–t90 min	0.926 (0.874, 0.981)	0.0088		
	Diff Insulin Conc. t60–t90 min	0.938 (0.882, 0.998)	0.0441		
	Diff Insulin Conc. t0–t120 min	0.952 (0.918, 0.988)	0.0098		
	Insulin Conc. t60 min	0.950 (0.912, 0.990)	0.0143		
	Insulin Conc. t90 min	0.925 (0.873, 0.980)	0.0087		
	Insulin Conc. t120 min	0.968 (0.941, 0.996)	0.0248		
	Cmax Insulin	0.968 (0.941, 0.995)	0.0210		
	Insulin iAUC t0–60 min	0.999 (0.998, 1.000)	0.0342		
	Insulin iAUC t0–90 min	0.999 (0.998, 1.000)	0.0077		
	Insulin iAUC t0–t120 min	0.999 (0.998, 1.000)	0.0055		
	Diff Insulin Conc. from fasting to Cmax	0.962 (0.932, 0.993)	0.0152		
	Diff C-peptide Conc. t0–t60 min	0.479 (0.267, 0.858)	0.0134		
	Diff C-peptide Conc. t0–t90 min	0.483 (0.277, 0.841)	0.0102		
	Diff C-peptide Conc. t0–t120 min	0.577 (0.372, 0.894)	0.0139		
	C-peptide Conc. t60 min	0.546 (0.335, 0.891)	0.0155		
	C-peptide Conc. t90 min	0.492 (0.283, 0.855)	0.0119		
	C-peptide Conc. t120 min	0.647 (0.447, 0.937)	0.0211		
	Cmax C-peptide	0.675 (0.473, 0.965)	0.0312		
	C-peptide iAUC t0–t60 min	0.984 (0.969, 0.998)	0.0303		
	C-peptide iAUC t0–t90 min	0.987 (0.976, 0.997)	0.0107		
	**C-peptide iAUC t0–t120 min**	**0.989 (0.982, 0.997)**	**0.0078**	**0.974 (0.956, 0.993)**	**0.0070**
	C-peptide iAUC t0–t240 min	0.996 (0.993, 0.999)	0.0163		
	C-peptide iAUC t0–t360 min	0.998 (0.996, 1.000)	0.0436		
	Diff C-peptide Conc. from fasting to Cmax	0.592 (0.381, 0.921)	0.0201		
	Average C-peptide Conc. t10–t360 min	0.500 (0.263, 0.951)	0.0346		
	Diff Glucose Conc. t0–t10 min	0.050 (0.003, 0.781)	0.0327		
	**Glucose Conc. t10 min**	**0.131 (0.020, 0.864)**	**0.0347**	**0.038 (0.002, 0.811)**	**0.0362**
	Glucose iAUC t0–t10 min	0.527 (0.259, 1.072)	0.0769		
Triacylglycerol	Diff Triglyceride Conc. t0–t240 min	3.872 (1.181, 12.692)	0.0254		
	Diff Triglyceride Conc. t120–t240 min	6.944 (1.376, 35.039)	0.0189		
	**Diff Triglyceride Conc. t60–t240 min**	**5.304 (1.449, 19.421)**	**0.0117**	**12.376 (1.734, 88.331)**	**0.0121**
	Diff Triglyceride Conc. t0–t360 min	2.891 (1.008, 8.295)	0.0483		
	Diff Triglyceride Conc. t60–t360 min	4.269 (1.268, 14.371)	0.0191		
Demographic characteristics	LDL-C (Fasting)	2.410 (0.898, 6.465)	0.0807		
	HDL-C (Fasting)	6.750 (0.753, 60.512)	0.0879		
	Cholesterol (Fasting)	2.194 (0.957, 5.031)	0.0635		

Odds ratios are expressed per standard deviation increase in each continuous risk factor. Highlighted variables are independent risk predictors of subclinical atherosclerosis admitted by stepwise selection into the multivariable logistic regression models. OR, Odds ratio; CI, Confidence interval; iAUC, incremental area under curve; Cmax, Peak concentration; LDLC3, Low-density lipoprotein cholesterol; HDLC4, High-density lipoprotein cholesterol.

Significant values are in bold.

Mediators of glucose metabolism were consistently associated with lower risk of SA, up to 2 h post-meal in females. Simultaneously high insulin and C-peptide responses suggest a robust pancreatic beta-cell function in disease-free females. The change in total plasma C-peptide concentrations from baseline to 2 h post-meal was the top predictor of SA for females, but this was not observed in males.

Substantial gender differences were also observed in markers of fat metabolism. The increase in total cholesterol concentration at 2-h post meal was the top predictor of SA within males, superseding the conventional FRS. Total cholesterol, as well as LDL-C and HDL-C were uniquely associated with lower risk of SA in males. Even though Table 3 suggests an association between fasting cholesterol with SA in females, this association was shown to be weak in the univariate logistic regression models (Table 5). Instead, there was a pronounced surge in triacylglycerol 4 h post-meal unique to SA females, that was not detected in the disease-free females nor the male population. Triacylglycerol concentrations in females with SA were consistently elevated throughout the postprandial period.

**Figure 1:**
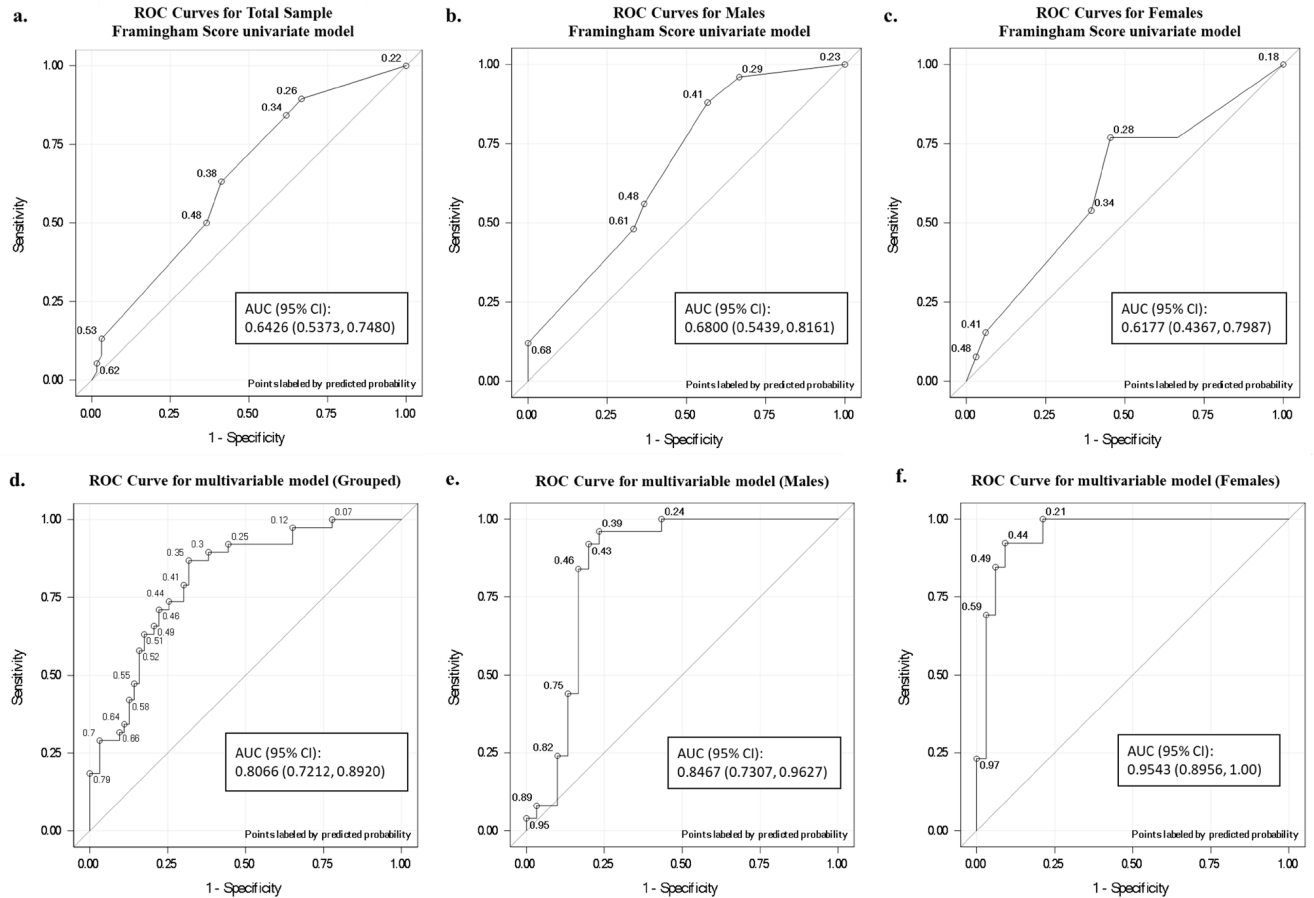
Comparison of ROC area under curve of Framingham scores (**a**–**c**) and our biomarker optimized models (**d**–**f**) to discriminate between presence and absence of subclinical atherosclerosis. Framingham scores predictive capability in the grouped analysis (**a**), in males (**b**) and females (**c**) showed better discriminative ability in males than females for subclinical atherosclerosis. The biomarker optimized models in the grouped analysis (**d**), in males (**e**) and females (**f**) consistently demonstrated improved predictive capability. CI, Confidence interval; AUC, area under curve.

The inflammatory marker TNFα displayed higher overall concentrations in both disease-free and SA males as compared to females. Additionally, FRS was found to be a candidate SA predictor specifically for males (Table 4). In both genders, FRS alone had unreliable predictive potential for atherosclerotic status similar to that of a random classifier, as reflected by the wide 95% confidence intervals (Fig. 1).

### Assessing predictive power of multivariable models

**Figure 2:**
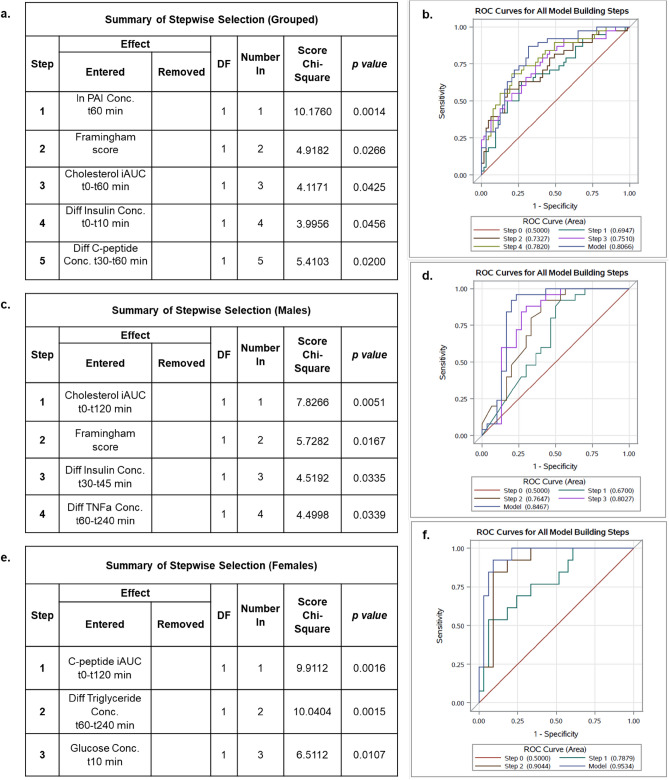
Summary of sequential entrance of variables into multivariable model and incremental improvements to prediction accuracy of subclinical atherosclerosis, reflected by the area under curve of ROC curve in the grouped analysis (**a**, **b**), in males (**c**, **d**) and females (**e**, **f**). TNFa, Tumor necrosis factor alpha; iAUC, incremental area under curve; PAI-1, Plasminogen activator inhibitor-1.

Next, we subjected these candidate predictors to a multivariable stepwise selection algorithm to create the final SA prediction model. Contrary to the initial model based on fasting biomarkers and anthropomorphic parameters, inclusion of the postprandial biomarkers improved FRS’s ability to reliably predict SA (Figs. 1, 2).

In the overall analysis, the ROC AUC for a linear combination of postprandial insulin, C-peptide, total cholesterol, PAI-1 and FRS was 0.8066 with 95% CI (0.7212, 0.8920) (Supplementary Fig. 3). At the 0.35 predictive probability optimized by Youden Index, PPV = 0.617 and NPV = 0.895.

**Figure 3:**
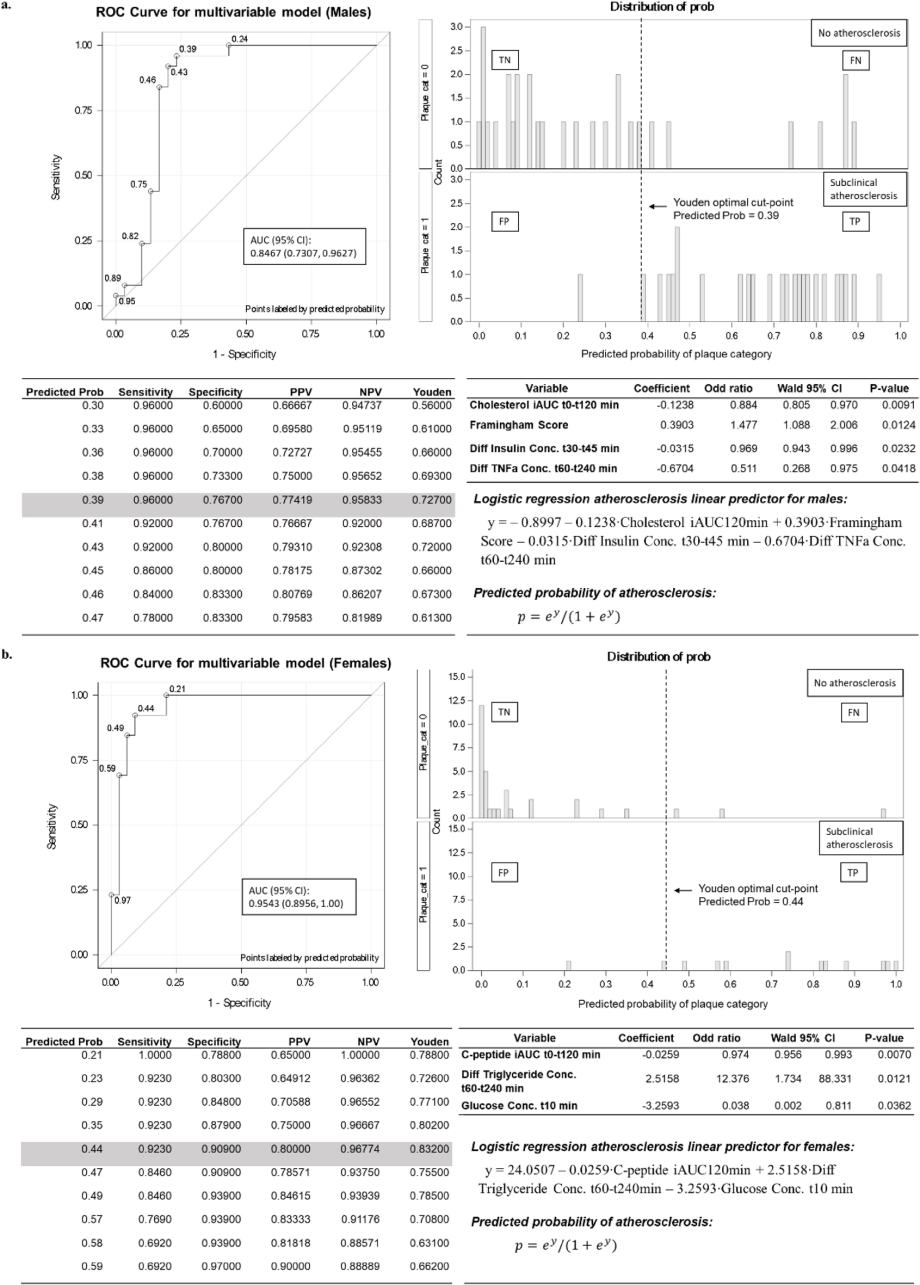
Summary of Logistic Regression and gender stratified ROC analysis results—A clinical tool for predicting risk of subclinical atherosclerosis in males (**a**) and females (**b**). ROC curve cut points with classification parameters, model coefficients, odds ratios and *p* values are shown. The ROC curve reflects prediction accuracy of multivariable model for presence of subclinical atherosclerosis. (**a**) Logistic regression atherosclerosis linear predictor for males: y = − 0.8997 − 0.1238∙Cholesterol iAUC120min + 0.3903∙Framingham Score − 0.0315∙Diff Insulin Conc. t30–t45 min − 0.6704∙Diff TNFa Conc. t60–t240 min (**b**) Logistic regression atherosclerosis linear predictor for females: y = 24.0507 − 0.0259∙C-peptide iAUC120min + 2.5158∙Diff Triglyceride Conc. t60-t240min – 3.2593∙Glucose Conc. t10 min. Predicted probability of atherosclerosis:〖p = e〗^y/(1 + e^y). TN, True negative; FN, False negative; FP, False positive; TP, True positive; PPV, Positive predictive value; NPV, Negative predictive value; CI, Confidence interval; AUC, Area under curve; TNFa, Tumor necrosis factor alpha; iAUC, incremental area under curve; PAI-1, Plasminogen activator inhibitor-1.

Subsequently, we computed two gender-specific models with different combinations of postprandial biomarkers. In males, the ROC AUC for a linear combination of change in insulin, TNFα, cholesterol and FRS improved to 0.847 with 95% CI (0.731, 0.963) (Fig. 3a). While TNFα and insulin have weak individual effects on SA categorization, additive effects of postprandial markers allowed them to contribute incrementally to the performance of the final model. Post-meal changes in cholesterol concentration at 2-h, insulin concentration from 30–45 min and TNFα concentration between 0.5 and 4 h were associated with 11.6%, 3.1% and 48.9% reduction in odds of SA. On the contrary, males with high FRS were 1.5-times more likely to have SA.

Amongst females, the ROC AUC for a linear combination of change in C-peptide, glucose and triacylglycerol improved to 0.954 with 95% CI (0.896, and 1.00) (Fig. 3b). Changes in post-meal C-peptide concentration at 2-h and glucose concentration at 10 min were both protective against SA by 2.6% and 96.2% respectively. Whereas females with an increase in postprandial triacylglycerol concentration between 1–4 h had concomitant 12.4-fold increased odds of SA. Absence of FRS in the female multivariable prediction model is congruent with literature findings on the limited prediction accuracy of FRS among females^8^. The male model had an improved PPV = 0.774 and NPV = 0.958, while the female model had an improved PPV = 0.800 and NPV = 0.967.

We used the Delong-Delong approach to compare the improvement in ROC AUC between our postprandial biomarker predictive model from the conventional Framingham score model. We found that our biomarker-optimized predictive models exhibited statistically significant improvements over the Framingham score model alone: *P* = 0.0021 for the overall sample analysis, *P* = 0.0241 for the male specific model and *P* = 0.0006 for the female specific model (Fig. 1).

### Cross-validation of gender-specific multivariable prediction models

**Figure 4:**
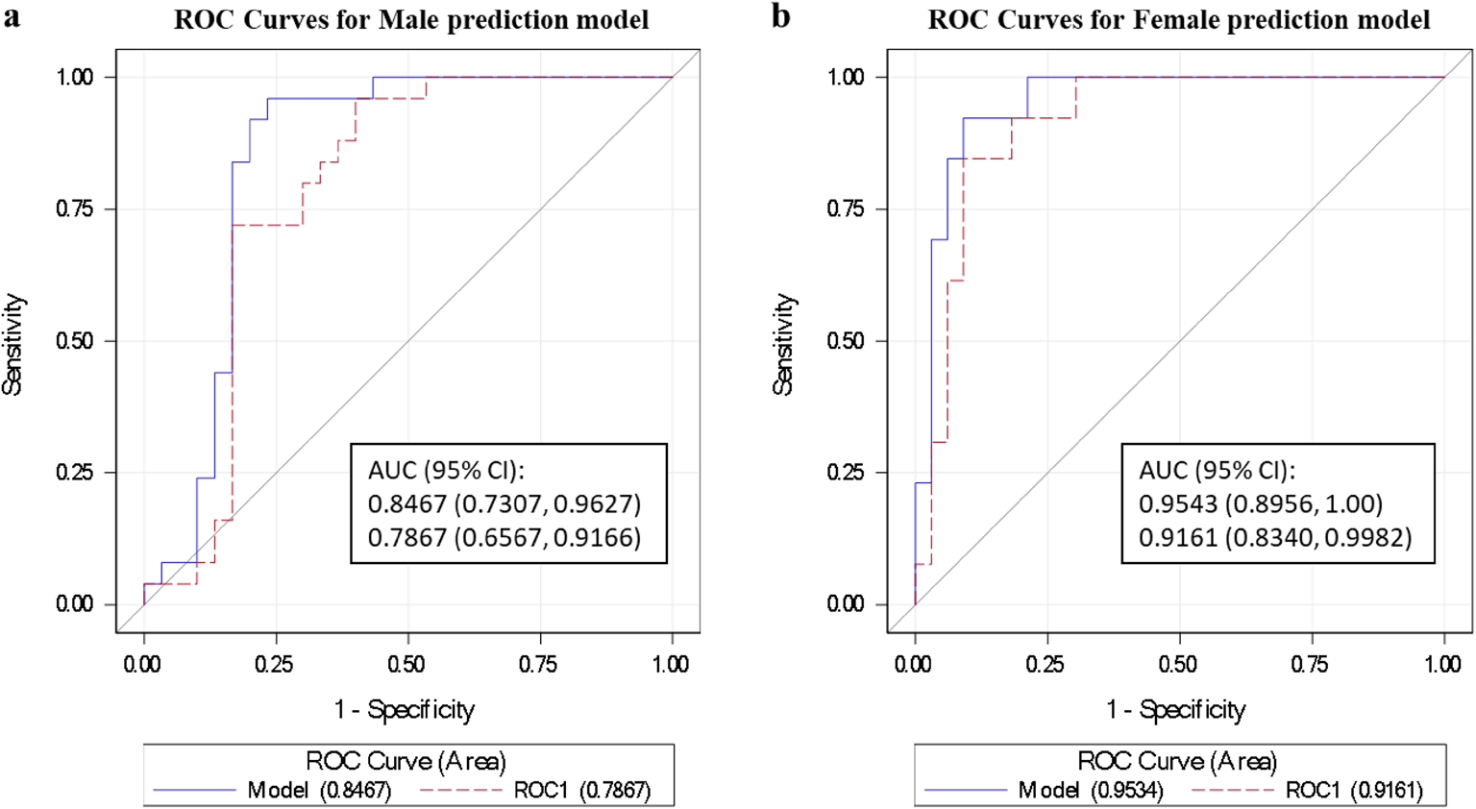
Evaluating subclinical atherosclerosis multivariable logistic regression models classifier output quality using cross-validation in male prediction model (**a**) and female prediction model (**b**). Model ROC curve in blue and cross-validated ROC curve in red, with respective AUC and 95% confidence intervals shown. CI, Confidence interval; AUC, area under curve.

Finally, we performed cross-validation to assess the performance of each gender-specific model. The ROC AUC after cross-validation is 0.787 (*P* = 0.0037) and 0.916 (*P* = 0.0216) in males and females, respectively (Fig. 4). In the male-specific model, PPV and NPV were revised to 0.783 and 0.781, while the female-specific model had a final PPV of 0.786 and NPV of 0.938. Classifier parameters revealed that the female-specific model accomplished better performance than the male-specific model in distinguishing those with SA.

## Discussion

The goal of this study was to develop a gender-specific SA risk estimator using a combination of clinical characteristics and postprandial biomarker responses to a mixed meal test in an Asian population at low risk of CHD. Our model yields improved disease estimates compared to FRS alone, if it were to be used as a singular metric (Fig. 1). Using a gender-specific approach also returned models with overall better predictive values and discrimination for SA individuals.

Our observations show that FRS remains a relevant positive predictor of atherosclerosis even in asymptomatic persons. However, the FRS model has proved to be inadequate in predicting SA in several large cross-sectional studies in American^30^ and European populations^7^. These studies reported presence of atherosclerosis in 69%^30^ and 58%^7^ amongst healthy persons with low FRS. More than one-third of our healthy Asian participants had SA despite a low FRS. In the absence of conventional CVRFs, other multifactorial etiologies may contribute to the early pathogenesis of atherosclerosis. Hence, constructing a postprandial risk estimator that includes subclinical measures of atherosclerosis may allow early clinical awareness of the atherosclerotic burden.

There are several well-described gender differences in the prevalence, progression and severity of atherosclerosis, but the underlying mechanisms are incompletely understood. Contributing reasons range from innate differences in vascular physiology, plaque morphology, sex hormones, risk factor profile and psychosocial factors^10^. Past studies have demonstrated the decisive role of menopause in the development of CVD in women, with an accelerated tenfold increase in CVD among post-menopausal women compared to a 4.6-fold increase among age-matched men^31^. In our study, HDL-C was significantly higher in our middle-aged, perimenopausal women than in men. With a favorable cholesterol profile built by pre-menopause physiology, female differences between the disease-free and SA group could be blunted. Lack of this natural advantage may explain the heightened association between postprandial cholesterol and SA exclusive to the male participants in our study. In our study sample, traditional CVRFs such as fasting triacylglycerol and cholesterol were not associated with SA in either gender. Instead, the postprandial concentrations of triacylglycerol and cholesterol were predictive of SA in females and males respectively. Earlier research reported that individuals with CVRFs often have an exaggerated triacylglycerol peak or delayed clearance, resulting in postprandial accumulation of atherogenic triacylglycerol-carrying lipoprotein particles^19^. An exaggerated postprandial triacylglycerol surge may also represent an abnormal metabolic response towards an oral fat load. This raises concerns for insulin resistance, which further predisposes to CVD^19^. Interestingly, our study did not find significant influence of lipoprotein cholesterol carriers, such as LDL-C and HDL-C, on atherosclerosis status. Only total cholesterol levels were independently associated with the presence of SA. Although LDL particles are in principle more atherogenic, low net concentration and minor between-group variability in a population with optimally low CVRFs could explain the failure to observe significant associations^32^. Fasting dyslipidemia and hypercholesterolemia may have a greater role to play in the setting of more advanced atherosclerosis.

Some conventional CVRFs are also known to have more importance for females. Our study observed a greater importance of indices of glucose metabolism in predicting SA in females. An early study on patients with diabetes by Stokes et al. demonstrated that deranged metabolic processing of glucose was a more significant CVRF for women than for men^33^. A comparison on influence of diabetes on the associated risk of myocardial infarction revealed an odds ratio of 1.6 in women compared to men^34^. Hence, recommendations for optimal glucose control should be emphasized for females to reduce CHD incidence.

Sexual dimorphism in early atherogenesis may also involve immune responses to hyperlipidemia. Our observations on inflammatory markers showed an association between high TNFα and reduced risk of SA among males. We failed to find significant association for other common markers of inflammation, including CRP and IL-6. It is possible that CRP and IL-6 are related to CVD outcomes but less related to subclinical disease^35^ or resilient to dietary challenges^36^. Pro-inflammatory cytokines, such as TNFα, are widely known to be atherogenic^21^, but studies on the efficacy of TNFα inhibitors to stall progression of SA in high-risk patients has been controversial^37,38^. Some studies also reported an association between presence of atherosclerotic plaque with TNFα receptors, but absence of association with TNFα itself^35^. Thus, the precise role of TNFα ligand in premature atherosclerotic plaques may be more complex and merits further study. We found a higher baseline and postprandial concentration of TNFα in males throughout the postprandial period. Prior large-scale investigations of carotid endarterectomy found males were significantly associated with increased inflammatory infiltrates in atherosclerotic plaques compared to age-matched females^34^. Recent reports demonstrate sex differences in levels of proatherogenic cytokines, including TNFα, but the mechanism behind this association remains poorly understood^39^. While the precise role of TNFα in this pro-inflammatory state and atherogenesis remains incompletely elucidated, evidence suggests high androgen exposure upregulates inflammatory genes^10^.

There are some limitations to the present study. Firstly, our data comprises of only Chinese subjects and the findings may not be generalizable to other ethnic groups in Singapore. A repeat of the data collection on a larger cohort would help to externally validate the SA risk prediction models. Secondly, our study model, based off peri-menopausal women aged 40 to 54, did not reflect their menopause status at the time of assessment, which may confound their subclinical atherosclerosis status. Thirdly, our cross-sectional study of SA association with diverse metabolites does not allow determination of temporal or causal relations. Therefore, longitudinal follow-ups of participants would allow us to draw conclusions on what proportion of SA plaques in a low-risk population transition into symptomatic onset of CVD and mortality in later decades. Nonetheless, corresponding extensive data collected from state-of-the-art vascular imaging technology with that of a simple commercially available mixed-meal test sets the groundwork for simplification of SA prediction, and provides useful insights into postprandial metabolic profile changes in relation to early stages of atherogenesis.

Despite the well-known delayed incidence of CVD in women, our study found that SA is equally prevalent in both genders. Physiological differences in postprandial lipid, glucose and inflammatory responses may then further discriminate the likelihood of each gender progressing into symptomatic CHD^10^. CVD is consistently the biggest contributor to Singapore’s burden of early death and disability (measured in disability-adjusted life years, or DALYs) at 14.3% of total DALYs from 1990 to 2017^2^. The financial impact of CVD involves both direct costs, such as hospitalization, medications, rehabilitation, outpatient care, as well as indirect costs pertaining to productivity losses, informal care, early mortality or retirement. The annual direct and indirect cost in Singapore totals to 8.2 billion USD, but 60% of these costs are attributed to modifiable risk factors^3^. This suggests that forewarning individuals to implement lifestyle changes or early clinical recommendations can help lower the financial toll of cardiovascular events. Environmental factors related to lifestyle, such as unhealthy dietary habits and lack of regular physical activity, undeniably play an important role in the development of CVD and thus make it largely preventable. Therefore, a diagnostic modality that supplements conventional risk stratification strategies would be economically important.

## Supplementary Information

Supplementary Information.
