# Supplementary material for: Gender differences in fasting and postprandial metabolic traits predictive of subclinical atherosclerosis in an asymptomatic Chinese population
Source: Sci Rep. 2022 Oct 7;12:16890. doi: 10.1038/s41598-022-20714-6 (PMC9546939; doi:10.1038/s41598-022-20714-6)
Supplement: Supplementary file 1 — Supplementary Information. [file 41598_2022_20714_MOESM1_ESM.pdf]

Gender differences in fasting and postprandial metabolic traits predictive of subclinical atherosclerosis in an asymptomatic Chinese population

Xinpeng Loh[1]*, Lijuan Sun[2]*, John Carson Allen[1], Hui Jen Goh[2], Siew Ching Kong[3], Weiting Huang[3], Cherlyn Ding[4], Nabil Bosco[4,5], Leonie Egli[5], Lucas Actis-Goretta[4], Faidon Magkos[6], Fabrizio Arigoni[4], Khung Keong Yeo[1,3], Melvin Khee-Shing Leow[1,2,7,8]

[1] Duke-NUS Medical School, Singapore, Singapore; [2] Singapore Institute for Clinical Sciences, Singapore; [3] National Heart Center Singapore, Singapore; [4] Nestlé Research Singapore Hub, Singapore; [5] Nestlé Institute of Health Sciences, Nestlé Research, Lausanne, Switzerland; [6] University of Copenhagen, Frederiksberg, Denmark; [7]Department of Endocrinology, Tan Tock Seng Hospital, Singapore; [8] Lee Kong Chian School of Medicine, Nanyang Technological University, Singapore

*Both authors contributed equally to the manuscript.

***Correspondence should be addressed to:**

Melvin Khee-Shing Leow, MBBS, MMed (Int Med), PhD, FAMS, FACP, FACE, FRCP (Edin), FRCPath. 30 Medical Drive, Singapore 117609. Tel: +65 6407 0105.

Email: melvin_leow@sics.a-star.edu.sg

APPENDIX

Supplementary Table 1: Demographic and clinical characteristics at baseline (fasting state) for subjects with no SA (Plaque 0), with SA at 1 vascular site (plaque 1), with SA at 2 vascular sites (plaque 2) and those with SA at 3 or more vascular sites (plaque 3).

VARIABLE	PLAQUE 0 (N=63)	PLAQUE 1 (N=27)	PLAQUE 2 (N=8)	PLAQUE 3 (N=3)	*P VALUE*
**Gender**					
Male	30 (29.7%)	18 (17.8%)	6 (5.9%)	1 (1.0%)	0.1752
Female	33 (32.7%)	9 (8.9%)	2 (2.0%)	2 (2.0%)	
**Smoking status**					
Non-smokers	62 (61.4%)	27 (26.7%)	7 (6.9%)	2 (2.0%)	**0.0264***
Smokers	1 (1.0%)	0	1 (1.0%)	1 (1.0%)	
**AGE**	46.91 ±4.40	48.33 ±3.82	50.0 ±3.51	51.67 ±2.08	0.0508
**BMI (KG/M2)**	23.54 ±2.92	23.92 ±2.90	22.07 ±2.50	22.78 ±1.75	0.2683
**DIASTOLIC BP (mmHg)**	74.92 ±10.27	77.33 ±9.24	75.88 ±10.45	72.0 ±9.17	0.5843
**SYSTOLIC BP (mmHg)**	115.37 ±11.14	121.63 ±15.79	117.25 ±12.05	110.67 ±10.41	0.2733
**WAIST CIRCUMFERENCE (CM)**	82.20 ±9.77	82.87 ±9.44	80.88 ±5.14	76.33 ±3.79	0.5248
**WEIGHT (KG)**	64.90 ±10.02	67.30 ±12.51	62.31 ±7.54	62.38 ±2.34	0.6414
**GLUCOSE (MMOL /L)**	5.03 ±0.36	5.10 ±0.42	5.10 ±0.47	4.70 ±0.30	0.3137
**LDL-C (MMOL/L)**	3.21 ±0.74	3.42 ±0.72	3.49 ±0.74	3.78 ±0.46	0.1862
**HDL-C (MMOL/L)**	1.55 ±0.27	1.51 ±0.38	1.56 ±0.51	1.41 ±0.38	0.7653
**TRIACYLGLYCEROL (MMOL/L)**	1.02 ±0.58	1.12 ±0.70	1.13 ±0.35	1.24 ±0.24	0.3133
**CHOLESTEROL (MMOL/L)**	5.22 ±0.87	5.44 ±0.85	5.56 ±0.92	5.76 ±0.45	0.1570
**FRAMINGHAM SCORE**	5.14 ±2.74	5.93 ±2.34	7.38 ±2.39	9.33 ±2.31	**0.0163***

Supplementary Table 2: Potential candidate predictors of subclinical atherosclerosis in grouped analysisError! Bookmark not defined. Error! Bookmark not defined. (n=101) assessed using univariable logistic regression.

**Category**	**Variables**	**Univariate logistic regression**		**Multivariable logistic regression**	
		**OR (95% CI)**	***p value***	**OR (95% CI)**	***p value***
**Thrombosis**	ln PAI-1 Conc. t0 min	4.194 (1.376, 12.780)	0.0117		
	**ln PAI-1 Conc. t60 min**	**6.715 (1.968, 22.905)**	**0.0024**	**6.755 (1.705, 26.761)**	**0.0065**
	ln PAI-1 Conc. t120 min	4.403 (1.431, 13.540)	0.0097		
	ln PAI-1 Conc. t360 min	4.901 (1.490, 16.117)	0.0089		
	ln Cmax PAI-1	11.357 (2.745, 46.993)	0.0008		
**Insulin sensitivity**Error! Bookmark not defined.	**Diff Insulin Conc. t0-t10 min**	**0.973 (0.942, 1.004)**	**0.0892**	**0.957 (0.923, 0.991)**	**0.0141**
	Diff Insulin Conc. t0-t60 min	0.983 (0.968, 0.998)	0.0309		
	Diff C-peptide Conc. t0-t60 min	0.818 (0.647, 1.034)	0.0923		
	**Diff C-peptide Conc. t30-t60 min**	**0.786 (0.595, 1.037)**	**0.0891**	**0.686 (0.494, 0.953)**	**0.0246**
	Diff Glucose Conc. t10-t20 min	2.166 (0.890, 5.273)	0.0887		
	ln Adiponectin Conc. t120 min	0.401 (0.138, 1.163)	0.0927		
	ln Adiponectin Conc. t360 min	0.338 (0.110, 1.038)	0.0582		
**Total cholesterol**	Diff Cholesterol Conc. t0-t60 min	0.076 (0.006, 0.927)	0.0435		
	Diff Cholesterol Conc. t0-t360 min	0.189 (0.029, 1.236)	0.0822		
	Diff Cholesterol Conc. from fasting to Cmax	0.052 (0.004, 0.734)	0.0286		
	Cholesterol Conc. t0 min	1.618 (0.941, 2.779)	0.0816		
	**Cholesterol iAUC t0-t60 min**	**0.892 (0.805, 0.988)**	**0.0281**	**0.857 (0.757, 0.969)**	**0.0143**
	Cholesterol iAUC t0-t120 min	0.958 (0.919, 0.999)	0.0437		
	Cholesterol iAUC t0-t240 min	0.979 (0.956, 1.002)	0.0764		
	Cholesterol iAUC t0-t360 min	0.987 (0.972, 1.002)	0.0812		
**Demographic characteristics**Error! Bookmark not defined.	Age	1.128 (1.018, 1.249)	0.0188 0.0214		
	**Framingham Score**	**1.213 (1.035, 1.422)**	**0.0172**	**1.262 (1.038, 1.533)**	**0.0195**

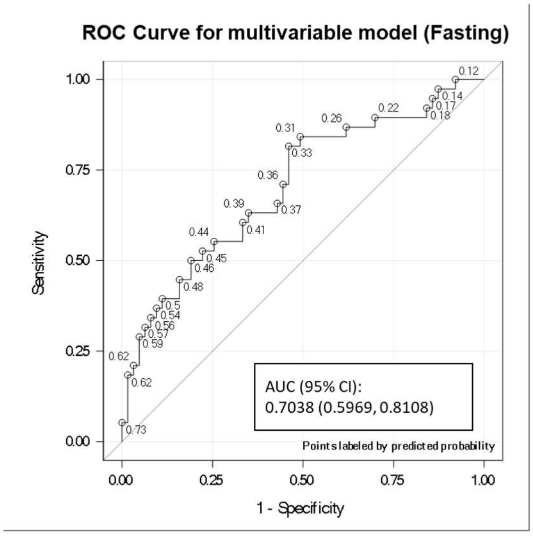

Predicted Prob	Sensitivity	Specificity	PPV	NPV	Youden
0.29	0.84200	0.42100	0.46718	0.81534	0.26300
0.30	0.84200	0.46000	0.48492	0.82848	0.30200
0.31	0.83333	0.50267	0.50265	0.83349	0.33600
0.32	0.81600	0.52400	0.50820	0.82500	0.34000
0.33	0.81600	0.54000	0.51667	0.82927	0.35600
0.35	0.76300	0.54000	0.49990	0.79098	0.30300
0.36	0.71100	0.54800	0.48653	0.75821	0.25900
0.37	0.66667	0.56100	0.47798	0.73618	0.22767

Variable	Coefficient	Odd ratio	Wald 95% CI		*p value*
PAI Conc. t0 min	0.000051	1.000	1.000	1.000	0.0156
Framingham Score	0.1927	1.212	1.026	1.433	0.0239

***Supplementary Figure 1*:** Summary of Logistic Regression and ROC grouped analysis using baseline (fasting) biomarkers to predict risk of subclinical atherosclerosis. ROC curve cut points with classification parameters, model coefficients, odds ratios and *p values* are shown. The ROC curve reflects prediction accuracy of multivariable model for presence of subclinical atherosclerosis in grouped analysis (n=101). Logistic regression atherosclerosis linear predictor in fasted state: y = –3.0450 + 0.000051·PAI Conc. t0 min + 0.1927·Framingham Score. Predicted probability of atherosclerosis: $p = e^y/(1 + e^y)$. TN, True negative; FN, False negative; FP, False positive; TP, True positive; PPV, Positive predictive value; NPV, Negative predictive value. Abbreviations: CI, Confidence interval; AUC, area under curve; PAI-1, Plasminogen activator inhibitor-1.

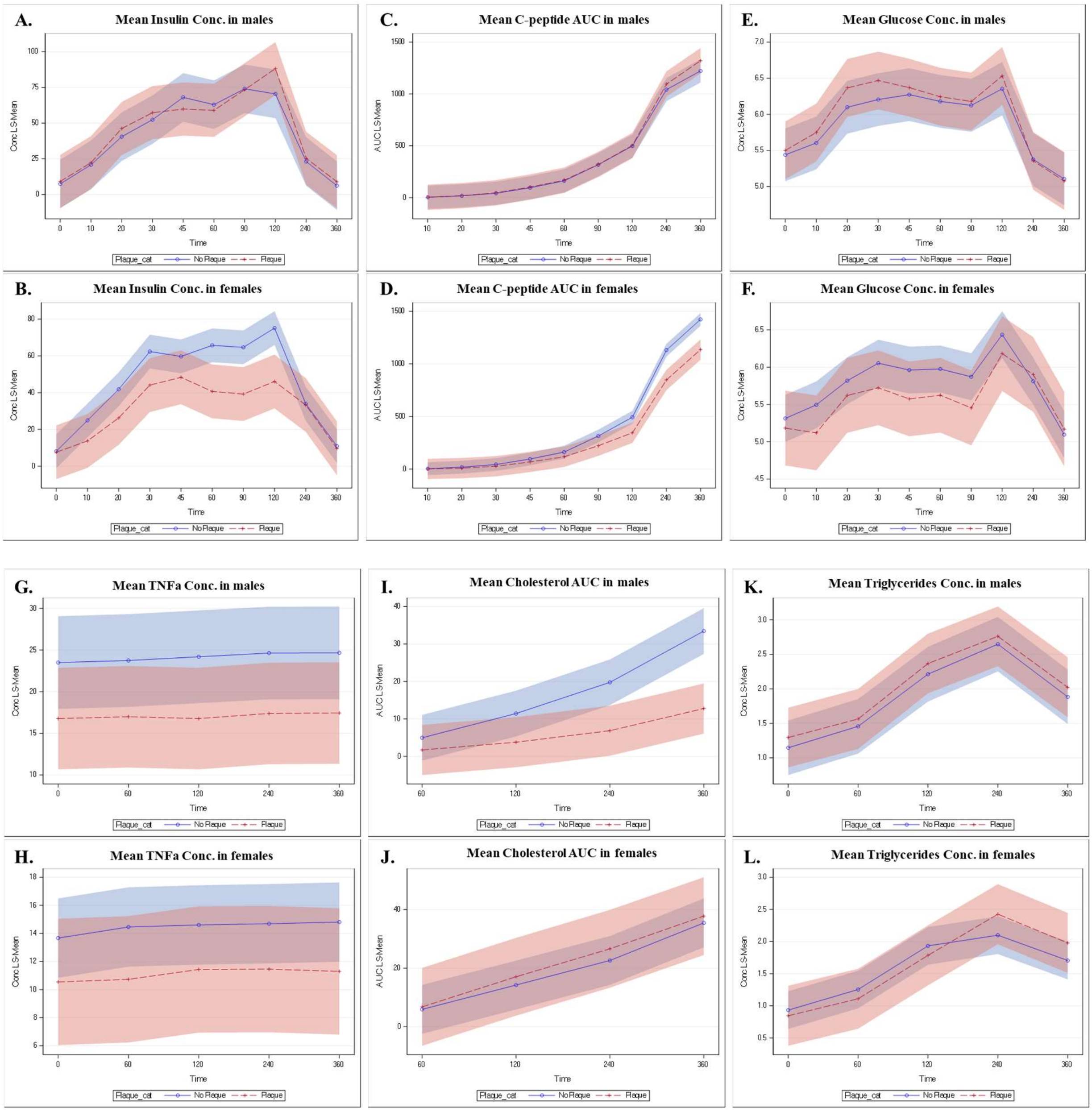

Supplementary Figure 2: Comparison of metabolic responses to mixed meal test in healthy males and females. Postprandial curves plotted as mean biomarkers concentration as a function of time, with 95% confidence interval for the top 6 predictor biomarkers in both genders: Insulin (A,B), C-peptide (C,D), Glucose (E,F), TNFα (G,H), Cholesterol (I,J) and Triacylglycerol (K,L). Abbreviations: TNFa, Tumor necrosis factor alpha; AUC, area under curve.

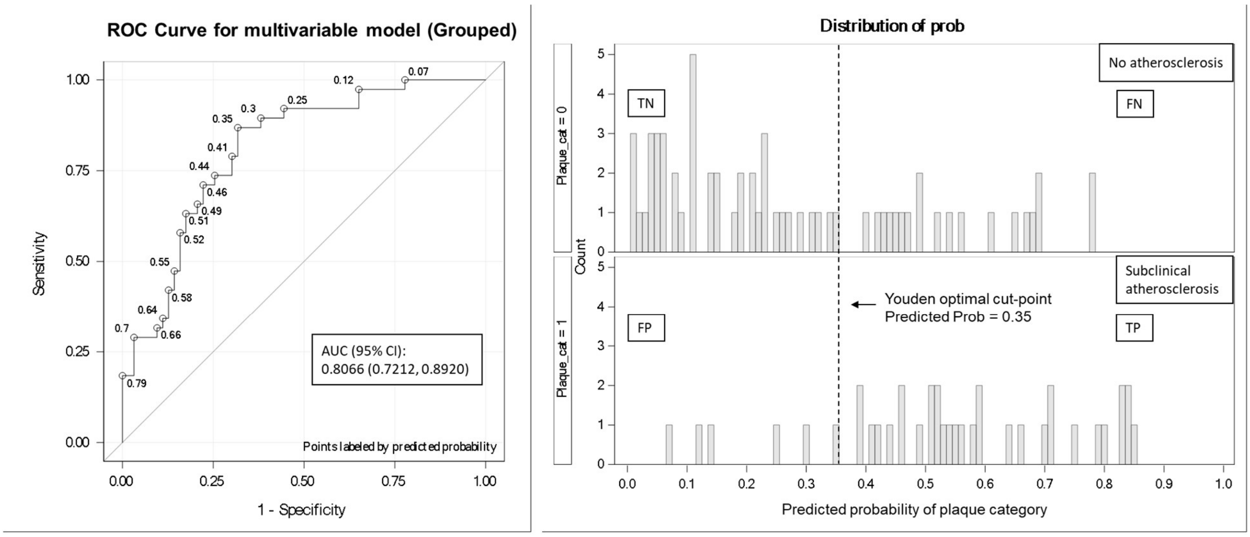

Predicted Prob	Sensitivity	Specificity	PPV	NPV	Youden
0.30	0.89500	0.61900	0.58621	0.90698	0.51400
0.31	0.86800	0.61900	0.57895	0.88636	0.48700
0.32	0.86800	0.63500	0.58929	0.88889	0.50300
0.34	0.86800	0.65100	0.60000	0.89130	0.51900
0.35	0.86800	0.67500	0.61688	0.89473	0.54300
0.39	0.82900	0.68300	0.61161	0.86878	0.51200
0.40	0.78900	0.68300	0.60000	0.84314	0.47200
0.41	0.78900	0.69800	0.61224	0.84615	0.48700
0.42	0.75000	0.69800	0.59996	0.82250	0.44800
0.43	0.73700	0.71400	0.60870	0.81818	0.45100
0.44	0.73700	0.73800	0.62929	0.82299	0.47500

Variable	Coefficient	Odd ratio	Wald 95% CI		P-value
**ln PAI Conc. t60 min**	1.9102	6.755	1.705	26.761	0.0065
**Framingham Score**	0.2324	1.262	1.038	1.533	0.0195
**Cholesterol iAUC t0-t60 min**	-0.1549	0.857	0.757	0.969	0.0143
**Diff Insulin Conc. t0-t10 min**	-0.0443	0.957	0.923	0.991	0.0141
**Diff C-peptide Conc. t30-t60 min**	-0.3769	0.686	0.494	0.953	0.0246

Logistic regression atherosclerosis linear predictor:

y = – 19.6409 + 1.9102·ln PAI Conc. t60 min + 0.2324·Framingham Score – 0.1549·Cholesterol iAUC t60 min – 0.0443·Diff Insulin Conc. t0-t10 min – 0.3769·Diff C-peptide Conc. t30-t60 min

Predicted probability of atherosclerosis:

$$p = e^y/(1 + e^y)$$

Supplementary Figure 3: Summary of Logistic Regression and ROC grouped analysis results - A clinical tool for predicting risk of subclinical atherosclerosis. ROC curve cut points with classification parameters, model coefficients, odds ratios and *p values* are shown. The ROC curve reflects prediction accuracy of multivariable model for presence of subclinical atherosclerosis in grouped analysis (n=101). Logistic regression atherosclerosis linear predictor of postprandial markers: y = – 19.6409 + 1.9102·ln PAI Conc. t60 min + 0.2324·Framingham Score – 0.1549·Cholesterol iAUC t60 min – 0.0443·Diff Insulin Conc. t0-t10 min – 0.3769·Diff C-peptide Conc. t30-t60 min. Predicted probability of atherosclerosis: $p = e^y/(1 + e^y)$ Abbreviations: CI, Confidence interval; iAUC, incremental area under curve; PAI-1, Plasminogen activator inhibitor-1; TN, True negative; FN, False negative; FP, False positive; TP, True positive; PPV, Positive predictive value; NPV, Negative predictive value.
